# Book Review: The New Public Health 3rd Edition

**DOI:** 10.3389/fpubh.2018.00265

**Published:** 2018-09-19

**Authors:** Mihajlo Jakovljevic, Katarina M. Janicijevic, Milos Stepovic

**Affiliations:** ^1^Department of Global Health, Faculty of Medical Sciences, Economics and Policy, University of Kragujevac, Kragujevac, Serbia; ^2^Department of Social medicine, Faculty of Medical Sciences, University of Kragujevac, Kragijevac, Serbia; ^3^Faculty of Medical Sciences, University of Kragujevac, Kragujevac, Serbia

**Keywords:** public health, new public health, twenty-first century, global health, population, public health systems

The book entitled “The New Public Health 3rd Edition” edited and authored by Professor Theodore Tulchinsky and Dr Elena Varavikova describes some of the key global health challenges and attainments of the twenty-first century. The New Public Health 3rd Edition consists of 16 chapters with the page count 912 and associated Index of terms. The first chapter describes the history of public health from the characteristics of prehistoric societies, medieval period, period of renaissance and revolutions up to the twentieth century (1). The contents of the second chapter of book include expanding to: the concept and evolution of public health, health and diseases, natural history of disease, society health, modes of prevention, demographic and epidemiologic transitions, interdependence of health services, defining public health, selective primary care, risk approach, case of action, political economy and health, social, behavioral and political sciences, health field concepts, value of medical care in public health, health targets, individual and community participation in health, human ecology and health promotion, defining public health standards, integrative approaches to public health, achievements, future and new public health. Remaining segments of the book are concepts devoted to: measuring, monitoring and evaluating the health of the population and include: the life expectancy, social epidemiology, sentinel events, burden of disease, years of potential life lost, standardization of rates, potential errors in measurement, screening of disease, notification of disease, special registries and reporting systems, disease classification, hospital information system, assessing the health of the individual, assessment of public health, health care financing and organization, and the health information to knowledge to policy of the public health (2).

Another book chapter devoted to communicable diseases deals with issues such as: public health and the control of communicable disease, nature of communicable disease, host-agent-environmental trial, and classifications of communicable disease, healthcare-associated infections, endemic and epidemic disease, control of communicable diseases, vaccine preventable diseases and vaccine programs. Given the huge and growing burden of NCDs in global morbidity and mortality patterns, chapter on non-communicable diseases and associated conditions is particularly important. It explains the ground causes of epidemiological transition, risk factors, role of individual and community preventive service in control of non-communicable diseases (3, 4). Family health chapter consists explanation on ways of recognizing specific health issues of family in different stages of life (5). Other chapters are: special community health needs; nutrition and food safety, environmental and occupational health and organization of public health systems.

Book contains an explanatory contribution introducing ground concepts of health economics. Relationship between priority of health choices and resource allocation constraints are well explained. The types of economic analysis and macroeconomic level of health financing, cost of illness, medical and hospital care and evaluative approaches to the New Public Health are all dealt with to a certain extent (6). Planning and managing health systems is a significant chapter which provides information about the role of management at all levels of health service and public health organization, ways how to apply management theory to health planning and public health, health system organization models and new organizational models (7). Chapter about national health systems describes major types of national health insurance and health service systems, assesses factors in health reform policies including variety of examples among developing countries and former Soviet countries (8). It compares national health systems and describes reforming national health systems from a time perspective.

Other parts of the book are: human resources for health, health technology of quality, law and ethics. The final chapter is about global health and new public health—where is written about global health situation and leading causes of disease and mortality in the world, priorities in global health, names of major organizations involved in international health and international standards, objectives and efforts in health, disease eradication, control, and health promotion. Reading the final chapter reminds us about important note of the global public health—One for all and all for one (9). The text contains numerous illustrations in the form of tables, graphics and pictures. It is worth mentioning appealing figure depicting an old telephone versus contemporary modern cell-phones. The latter are recognized as the practical tools for the public health services - …“*Looking back helps in looking ahead*.”

Various aspects of the current public health were covered and strategies to cope with these issues were analyzed. Book summarizes the old and the new information on global public health challenges and presents significant source of information for wide audience. The New Public Health has at first been intended for use to the students of medicine, social medicine, and interdisciplinary health sciences to serve as higher education material and cover an existing knowledge gap in applied health and social sciences. The book may serve as standard textbook in graduate and postgraduate public health curricula. This book is approachable, instructive, practical, and timely.

It would have been most certainly of substantial benefit if authors had considered discussing about actual problems of paperwork issues that are very common to health systems in transition and among rapidly developing Emerging nations serving as the engine of global economy for a long time. Use of The New Public Health could facilitate capacity building process among public health professionals worldwide. Regions and countries find themselves in diverse stages of historical evolution of their health systems. Therefore, they expose an array of different needs and vulnerabilities in the process of medical facilities establishment. It is an intersectional and interdisciplinary application of social policy, health promotion, preventive and curative health services, all of which are vital to sustain and improve New Public Health for communities worldwide. Public health as a traditional field of interdisciplinary science has brought fruitful textbook development in all world languages. Variety of prominent contributions in the field covered most of the traditional public health concepts and disciplines. However unique to New Public Health is its comprehensive presentation of XXI century challenges such as global population aging, large scale migrations, megacities and accelerated urbanization among developing nations, climate change etc. Therefore, we believe this book is worthy literature addition to academic libraries and lay audience alike.

## Author contributions

MJ, KJ, and MS are all responsible for the design and conception, writing the article, critical revision of the article, final approval of the manuscript, and overall responsibility.

### Conflict of interest statement

The authors declare that the research was conducted in the absence of any commercial or financial relationships that could be construed as a potential conflict of interest.
